# Spondyloarthritis and inflammatory bowel disease: towards a more severe and refractory disease phenotype

**DOI:** 10.1016/j.ero.2025.12.001

**Published:** 2026-01-12

**Authors:** Juliane Michel, Clément Prati, Lucine Vuitton, Charline Vauchy, Eric Toussirot, Frederic Mauny, Marc Puyraveau, Mickaël Chouk, Olivier Fakih, Daniel Wendling, Frank Verhoeven

**Affiliations:** 1 Université Marie et Louis Pasteur, Service de Rhumatologie, CHU de Besançon, 25000 Besançon, France; 2 Université Marie et Louis Pasteur, UFR Santé, UMR1098, EFS, INSERM, Besançon, France; 3 Université Marie et Louis Pasteur, Service de Gastro-entrologie, CHU de Besançon, 25000 Besançon, France; 4 Université Marie et Louis Pasteur, Centre investigation clinique INSERM CIC-1431, CHU de Besançon, 25000 Besançon, France

## Abstract

**Objectives:**

The coexistence of inflammatory bowel disease (IBD) and spondyloarthritis (SpA) poses significant diagnostic and therapeutic challenges. This study aimed to compare clinical characteristics and treatment profiles of patients with both SpA and IBD (SpA/IBD) to those with SpA or IBD alone.

**Methods:**

We performed a single-centre observational study including 62 consecutive patients with SpA/IBD followed between 2019 and 2022. Patients met Assessment of SpondyloArthritis International Society 2009 criteria for SpA and had confirmed IBD. Demographics, clinical features, imaging, and biologic disease-modifying antirheumatic drug (bDMARD) usage were collected. For comparison, we included 100 patients with a single diagnosis of SpA or IBD, representing the Maladies Inflammatoires SysTémIques Chroniques cohort enrolled over the same period.

**Results:**

Among patients with SpA/IBD (51% male, 67% HLA-B27+), IBD was more often diagnosed first. Compared with SpA alone, patients with SpA/IBD had more psoriasis (27% vs 17%, *P* = .04), uveitis (27% vs 18%, *P* = .08), and smoking (66% vs 44.9%, *P* = .01), but lower HLA-B27 positivity (63% vs 80%, *P* = .002). They also received more bDMARDs (2.8 ± 1.7 vs 2.0 ± 1.15, *P* = .01). Compared with IBD alone, patients with SpA/IBD had higher rates of uveitis (27% vs 1%, *P* = .08), psoriasis (27% vs 20%, *P* = .04), smoking (66% vs 44.9%, *P* = .01), and greater disease activity (Harvey-Bradshaw Index at diagnosis 8.2 ± 5.7 vs 2.6 ± 3.1, *P* = .0006). Notably, 31.75% met criteria for difficult-to-manage disease, exceeding the 9% reported in SpA monodiagnosis. Combination bDMARD therapy was required in 9.7% of patients with SpA/IBD, significantly more than in single-diagnosis groups (*P* < .05).

**Conclusions:**

Patients with SpA/IBD exhibit more severe disease and therapeutic challenges, highlighting the need for tailored management and consideration of earlier combination biologic therapies.

**WHAT IS ALREADY KNOWN ON THIS TOPIC**

• The coexistence of inflammatory bowel disease (IBD) and spondyloarthritis (SpA) represents a therapeutic challenge with overlapping and divergent treatment goals.

**WHAT THIS STUDY ADDS**

• Patients with SpA/IBD have higher disease activity on both rheumatologic and intestinal fronts.
• They are more frequently difficult to manage than patients with either SpA or IBD alone.

**HOW THIS STUDY MIGHT AFFECT RESEARCH, PRACTICE OR POLICY**

• Patients with SpA/IBD require more tailored management strategies, and earlier consideration of biologic disease-modifying antirheumatic drug combinations may be warranted.

Alt-text: Unlabelled box dummy alt text

## INTRODUCTION

Axial spondyloarthritis (axSpA) is a chronic inflammatory rheumatic and musculoskeletal disease with a prevalence of 0.15% for ankylosing spondylitis and 0.3% for the broader spondyloarthritis (SpA) spectrum in Metropolitan France [1,2]. SpA is frequently associated with extramusculoskeletal manifestations, including uveitis, psoriasis, and inflammatory bowel disease (IBD), the latter comprising ulcerative colitis (UC) and Crohn’s disease (CD). Among these comorbidities, IBD is of particular interest because of its shared pathophysiological mechanisms with SpA, notably involving the gut/enthesis axis. This axis links intestinal dysbiosis and increased gut permeability to bacterial translocation, subsequently activating dendritic cells and triggering the interleukin (IL)-23/Th17 inflammatory pathway [[3], [4], [5]]. Asymptomatic gut inflammation affects nearly 60% of patients with SpA [6], underscoring the relevance of subclinical digestive involvement. The prevalence of clinically overt IBD in patients with early SpA was approximately 10% in the DESIR cohort, with a 5-year incidence of 0.95 per 100 patient-years [7]. Conversely, among patients with IBD, the prevalence of axial and peripheral SpA has been estimated at 5% and 16%, respectively [8]. The significance of this association is recognised in multiple classification criteria for SpA [[9], [10], [11]].

Joint involvement in patients with coexisting SpA and IBD appears to differ from that in isolated axSpA. In the Assessment of SpondyloArthritis International Society (ASAS) peripheral SpA (perSpA) study, IBD prevalence was similar in patients with axSpA and perSpA (4.4% vs 4.7%) [12]. In the subset of patients with IBD-related SpA, axial involvement was present in 58%, with lower HLA-B27 positivity (31.8% vs 78.8%) and more frequent peripheral arthritis (77% vs 36%), dactylitis (12.5% vs 6%), and less frequent psoriatic arthritis (3% vs 5.7%). Despite clear pathophysiological links, studies focusing on the SpA+IBD overlap population remain scarce. Notably, Luchetti et al [13] recently reported increased bacterial translocation in patients with concomitant SpA and IBD compared with those with IBD alone, suggesting a more pronounced inflammatory state. However, the clinical spectrum and burden of disease in patients with both conditions remain incompletely characterised.

This study aimed to describe the clinical characteristics of patients with a dual diagnosis of SpA and IBD, and to compare them with patients diagnosed with either condition alone

## METHODS

### Patients

Patients were identified in the Maladies Inflammatoires SysTémIques Chroniques (MISTIC) cohort, which is a monocentre, prospective real-world cohort. This cohort is being conducted at the University Hospital of Besançon, France, in the Rheumatology and Gastroenterology departments. This cohort began in January 2019 and is still ongoing. The aim is to collect sociodemographic, clinical activity, biological, imaging, and therapeutic data from patients followed up in these departments for inflammatory rheumatic diseases (rheumatoid arthritis, SpA, and psoriatic arthritis) or IBD (CD and UC). Follow-up is carried out in a standardised form by the rheumatologist and/or gastroenterologist.

For this study, we included patients from the MISTIC cohort who were followed between January 2019 and December 2022. We selected all consecutive patients with newly diagnosed SpA, or under follow-up for SpA, who met the ASAS 2009 classification criteria [9] and had a confirmed diagnosis of IBD by a gastroenterologist. To allow meaningful comparisons and ensure sufficient statistical power, we also included a representative sample of 100 patients with a single diagnosis of SpA or IBD, enrolled over the same period.

MISTIC cohort was registered by Besançon University Hospital (registration number P/2019/433; NCT04191395). Patients received information about the use of their medical data and did not object to the study before its start in accordance to the local Good Clinical Practice guidelines and with the Declaration of Helsinki. All participants were provided informed consent during the study. Clinical Research and Innovation Department of the Besançon University Hospital approved the experimental protocol. As a retrospective study, ethical review and approval were not required for the study in accordance with the local legislation and institutional requirements.

### Data collection

Data were retrospectively extracted from medical records, including demographics (age, sex, occupation, and smoking status). For patients with SpA, the following information was collected: date of diagnosis, human leukocyte antigen B27 (HLA-B27) status, presence of psoriasis, uveitis, and dactylitis; SpA phenotype (axial, peripheral, or mixed). Evidence of structural damage to the spine or sacroiliac joints based on radiographs, computed tomography, or magnetic resonance imaging was assessed with local evaluation.

Disease activity was assessed using the Ankylosing Spondylitis Disease Activity Score (ASDAS) with C-reactive protein (CRP) and the Bath Ankylosing Spondylitis Disease Activity Index, recorded from the time of codiagnosis to the most recent follow-up visit. For IBD, we recorded the subtype (CD or UC), disease activity scores, Harvey-Bradshaw Index (HBI) for CD and Mayo score (Ulcerative colitis disease activity index) for UC, and remission status at last visit.

All pharmacological treatments were recorded, including nonsteroidal anti-inflammatory drugs and biologic disease-modifying antirheumatic drugs (bDMARDs). We documented treatment duration, discontinuation reasons, and combination therapies, when applicable.

We applied a previously validated definition of difficult-to-manage (D2M) disease [14]:

1. Failure of ≥2 bDMARDs or targeted synthetic DMARDs (tsDMARDs) with different modes of action.
2. Insufficient disease control (any of: (i) high disease activity (ASDAS ≥ 2.1), (ii) signs of active disease (eg, peripheral manifestations) or (iii) reduced health-related quality of life).
3. Problematic management situation in the patient’s or rheumatologist’s perspective.

Patients with coexisting SpA and IBD were compared with patients with either condition alone, derived from the same MISTIC cohort.

### Statistical analysis

Quantitative variables are presented as means ± SD and were compared using the Student’s *t*-test or Mann-Whitney *U* test, as appropriate. Categorical variables are expressed as numbers and percentages and were compared using the Chi-squared test. Pearson’s or Spearman’s correlation coefficients were used to assess associations between patient characteristics and disease activity scores. Statistical significance was set at *P* < .05. All analyses were performed using SAS software, version 9.4 (SAS Institute Inc).

## RESULTS

### Clinical characteristics of patients with SpA and IBD

**Figure 1:**
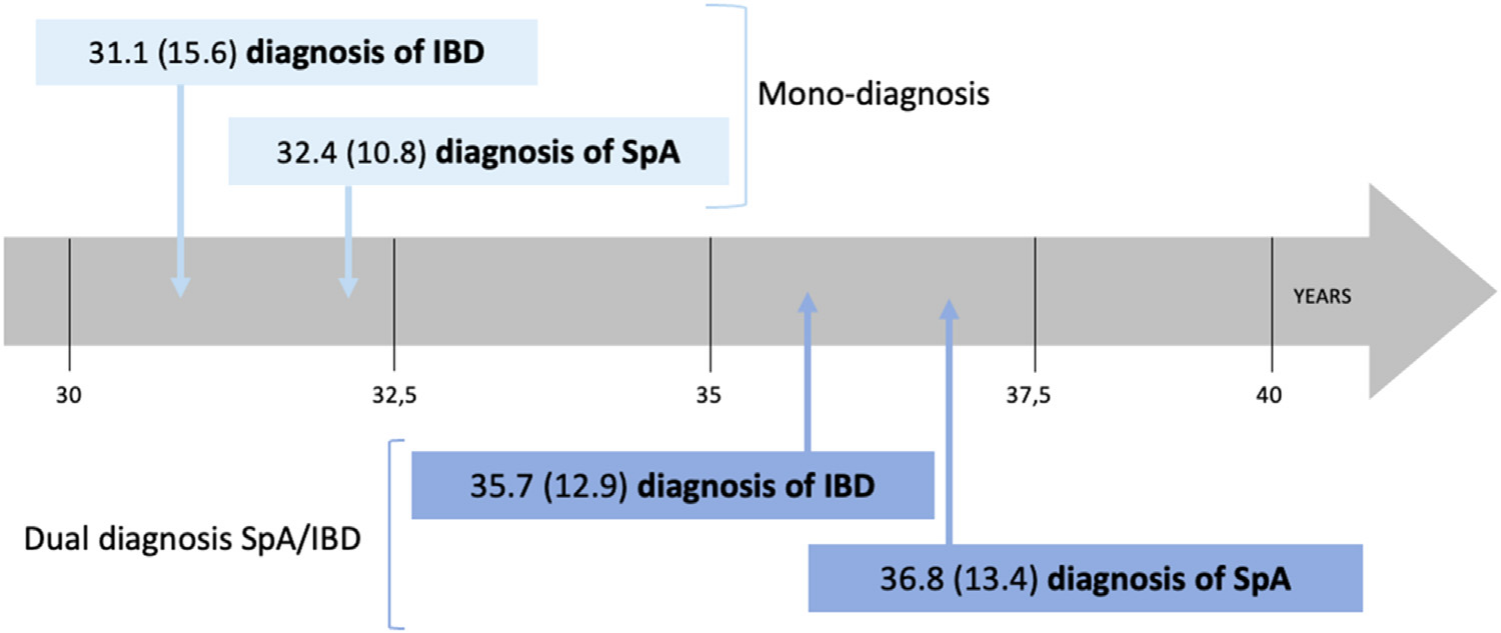
Order of appearance of SpA and IBD diagnoses. Data above the line refer to mono diagnosis. Those below concern the population with dual diagnoses. Data represent mean (SD). IBD, inflammatory bowel disease; SpA, spondyloarthritis.

The characteristics of the study population are summarised in Table 1. A total of 62 patients with a dual diagnosis of SpA and IBD were included; 51% were male and 67% were HLA-B27 positive. In most cases (61%, n = 38), IBD preceded the diagnosis of SpA. Among patients with IBD, CD was the most common subtype (68%), with a mean age at diagnosis of 35.7 ± 12.9 years (Fig 1).

**Table 1:** Clinical characterictics of patients with SpA and IBD.

Characterictics	Global population (n = 62)	SpA first (n = 24)	IBD first (n = 38)	*P* value
Sex (% male)	51%	62%	39%	.077
Smoking	66%	64%	68%	.76
Physical work	20%	19%	21%	1
Age at diagnosis of IBD	**35.7 ± 12.9**	**31.9 ± 11.8**	**38.6 ± 13.9**	**.043**
Age at diagnosis of SpA	**36.8 ± 13.4**	**29.8 ± 11.6**	**41.6 ± 12.6**	**<.001**
Disease duration IBD (y)	**17.1 ± 10.5**	**11.9 ± 5.24**	**20.0 ± 11.4**	**<.001**
Disease duration SpA (y)	**14.0 ± 9.84**	**19.3 ± 8.14**	**10.1 ± 8.44**	**<.001**
HLA B27+	**63%**	**83%**	**42%**	**<.01**
Crohn’s disease	68%	71%	66%	.38
axSpA	95%	96%	95%	1
Sacroiliitis (Rx)	**55%**	**86%**	**34%**	**<.001**
Sacroiliitis (MRI)	50%	81%	79%	1
Anterior or posterior spondylitis (MRI)	50%	55%	50%	.81
Syndesmophytes (Rx)	**27%**	**44%**	**13%**	**.036**
Psoriasis	24%	17%	29%	.27
Uveitis	**24%**	**35%**	**16%**	**.087**
Number of bDMARDs	2.8 ± 1.7	3.12 ± 2.03	2.61 ± 1.55	.39
Association bDMARDs	9.5 %	4.2%	13%	.39

Note: Bold values are significant.

axSpA, axial spondyloarthritis; bDMARD, biological disease-modifying antirheumatic drug; HLA, human leukocytic antigen; IBD, inflammatory bowel disease; MRI, magnetic resonance imaging; Rx, X-ray; SpA, spondyloarthritis.

Regarding the SpA phenotype, the vast majority (95%) had axial involvement, of which 55% met the criteria for radiographic axSpA. The mean age at SpA diagnosis was 36.8 ± 13.4 years. Extramusculoskeletal manifestations included psoriasis (24%) and uveitis (24%). Notably, 66% of patients were active smokers, and 20% had a physically demanding occupation.

Patients had received a mean of 3 bDMARDs during follow-up, and 6 patients (9.7%) were treated with a combination of biologic agents. Among patients who developed SpA in the context of IBD, 16 (42%) were already on bDMARDs (15 treated with tumor necrosis factor (TNF) inhibitors and 1 treated with vedolizumab), and conversely, among patients who developed IBD in the context of SpA, 12 (50%) were already on bDMARDs (12 treated with TNF inhibitors), with no significant difference between the 2 situations.

When stratified by the sequence of diagnoses (SpA-first vs IBD-first), significant differences emerged. In patients in whom SpA was diagnosed first (n = 24), the age at SpA diagnosis was significantly lower (29.8 ± 11.6 years vs 41.6 ± 12.6 years, *P* < .001), as was the age at subsequent IBD diagnosis (31.9 ± 11.8 years vs 38.6 ± 13 years, *P* = .043).

HLA-B27 positivity was significantly more common in the SpA-first group (83% vs 42%, *P* < .01). Psoriasis (17% vs 29%, *P* = .27) and uveitis (35% vs 16%, *P* = .087) showed trends but did not reach statistical significance.

Importantly, patients with SpA as the initial diagnosis had more frequent radiographic damage, with 86% presenting sacroiliitis (≥grade 2) compared with 34% in the IBD-first group (*P* < .001), and 44% had syndesmophytes vs 13% (*P* = .036).

### Comparison with patients with a single diagnosis

**Figure 2:**
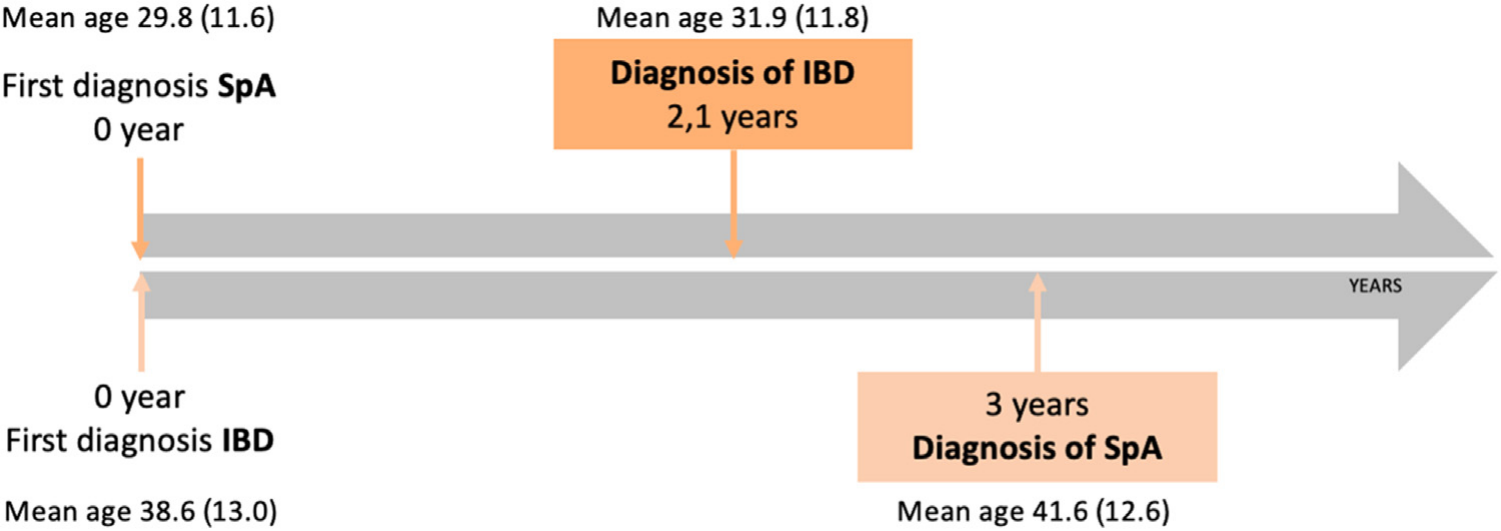
Timing of onset of SpA and IBD relative to each other. Data are presented as horizontal line; the top line represents SpA and the bottom line IBD. Data represent mean (SD). IBD, inflammatory bowel disease; SpA, spondyloarthritis.

Comparative data with monodiagnosis patients from the MISTIC cohort are presented in Table 2, and summarised in Figure 2.

**Table 2:** Comparison of patients with a dual diagnosis of SpA-IBD vs patients with a single diagnosis of IBD alone or SpA alone

Characterictics	SpA/IBD (n = 62)	IBD alone (n = 100)	*P* value	SpA alone (n = 100)	*P* value
Sex (% male)	51%	54%	.5	58%	.5
Smoking	**66%**	**51.5%**	**.01**	**44.9%**	**.01**
Age at diagnosis of IBD	**35.7 ± 12.9**	**31.1 ± 15.6**	**.05**	**-**	**-**
Disease duration IBD (y)	17.1 ± 10.5	14.5 ± 10.6	.13	-	-
Age at diagnosis of SpA	**36.8 ± 13.4**			**32.4 ± 10.8**	**.02**
Disease duration SpA (y)	14.0 ± 9.84			15.2 ± 11.7	.17
HLA B27+	**63%**	-	-	**80%**	**.002**
axSpA	95%	-	-		**1**
Crohn’s disease	68%	68%	1	**-**	**-**
Psoriasis	**24%**	**20%**	**.005**	**17%**	**.04**
Uveitis	**24%**	**1%**	**.087**	**18%**	**.087**
Number of bDMARDs	**2.8 ± 1.7**	**1.65 ± 0.8**	**.01**	**2 ± 1.15**	**.01**
Association bDMARDs	9.5 %	0%	**.03**	0%	**.03**
HBI score at diagnosis	**8.2 ± 5.7**	**2.6 ± 3.1**	**.0006**	-	-
HBI score at last consultation	**4.2 ± 3.4**	**2.4 ± 2.5**	**.0074**	**-**	**-**
UCDAI score at diagnosis		1.2 ± 1.6		-	**-**
UCDAI score at last consultation	**4.2 ± 3.4**	**0.6 ± 1.2**	**.02**	-	-
Crohn remission	**48.3%**	**85.1%**	**.0001**	**-**	**-**
UC remission	**37.5%**	**80.6%**	**.0001**	**-**	**-**
BASDAI at diagnosis	4.67 ± 1.92	-	-	3.99 ± 2.38	.11
ASDAS CRP at diagnosis	**2.86 ± 1.03**	**-**	**-**	**2.1 ± 1.06**	**.003**
CRP at SpA diagnosis	**9 ± 2.3**	**-**	**-**	**20.8 ± 5**	**.0001**

ASDAS CRP, Ankylosing Spondylitis Disease Activity Score with CRP; axSpA, axial spondyloarthritis; BASDAI, Bath Ankylosing Spondylitis Disease Activity Index; bDMARD, biological disease-modifying antirheumatic drug; CRP, C-reactive protein; HBI, Harvey-Bradshaw Index; HLA, human leukocytic antigen; IBD, inflammatory bowel disease; Rx, X-ray; SpA, spondyloarthritis; UC, ulcerative colitis; UCDAI, ulcerative colitis activity index.

Comparisons were performed pairwise (dual diagnosis SpA-IBD vs IBD alone; dual diagnosis SpA-IBD vs SpA alone).

When compared with patients with a single diagnosis of SpA, those with a codiagnosis of SpA and IBD were significantly older at diagnosis and more frequently current or former smokers. The prevalence of HLA-B27 positivity was lower in the codiagnosis group. In contrast, psoriasis was significantly more frequent and uveitis tended to be more prevalent, although not statistically significant. Patients with codiagnosis had been exposed to a greater number of bDMARDs, and disease activity scores and CRP levels were significantly higher at the time of SpA diagnosis.

When compared with patients with IBD alone, those with a codiagnosis of IBD-SpA were again older at diagnosis and more often smokers (current or past). They more frequently exhibited extraintestinal manifestations, particularly uveitis and psoriasis. Use of biologic therapy was also significantly more common in this group.

Importantly, patients with dual diagnosis had higher IBD activity scores (both HBI for CD and Mayo-ulcerative colitis disease activity index for UC) at the time of IBD diagnosis and at last follow-up, compared with patients with IBD alone. Clinical remission rates at last follow-up were significantly lower in the codiagnosis group. Regarding disease refractoriness, 20 patients (32.3%) met criteria for D2M disease, having received at least 2 bDMARDs with distinct mechanisms of action.

Finally, combination bDMARD therapy was used in 6 patients (9.7%) with dual diagnosis, whereas none in the monodiagnosis groups received such combination therapy (*P* = .003). Among these, 4 patients were treated with a combination of anti-TNF and vedolizumab, and 2 patients received a combination of anti-IL12/23 and vedolizumab

### Chronology of occurrence

When SpA was the first diagnosis (38,71%), the mean age at diagnosis was 29.8 years (±11.6), and the mean time to onset of IBD was 2.1 years, with a mean age at second diagnosis of 31.9 years (±11.8).

Conversely, when IBD was diagnosed first (61.29%), the mean age at diagnosis was 38.6 years (±13.0). The mean time to second diagnosis was 3 years, with a mean age at second diagnosis of 41.6 years (±12.6). These results are shown in Figure 2.

## DISCUSSION

To our knowledge, this is the first study specifically addressing patients with a dual diagnosis of SpA and IBD, with a particular focus on the temporal sequence of disease onset. Although the overall demographic profile of our cohort seems similar to that of patients with SpA alone, especially regarding age, sex distribution, and the presence of extramusculoskeletal manifestations, important differences emerged depending on which disease was diagnosed first. Notably, the prevalence of HLA-B27 in our dual-diagnosis population was lower (60%) than what is typically observed in classic axSpA cohorts (85%-90%), a finding in line with previous reports [8]. This supports the notion that SpA associated with IBD may represent a distinct phenotype.

In patients for whom SpA preceded IBD, the phenotype was consistent with classic axial SpA comparable to established cohorts [15]. Interestingly, we observed a higher prevalence of uveitis (35%) and psoriasis (17%) than previously reported in axSpA populations (20%-25% and 5%-9%, respectively) [16]. These findings may reflect an inflammatory burden that is globally higher in this subgroup. Previous studies have shown that elevated disease activity may be associated with increased risk of developing extramusculoskeletal manifestations [17], [18]. Conversely, in patients for whom IBD preceded SpA, the phenotype appeared closer to that of classic IBD cohorts. In this group, HLA-B27 prevalence dropped to 42%, in line with literature estimates around 30% [19], and uveitis prevalence was lower (16%), consistent with previously reported ranges in IBD cohorts (4%-12%) [20]. However, psoriasis prevalence was unexpectedly high (29%), compared with the 2% to 10% typically reported in epidemiological IBD studies [21]. Whether this reflects a shared immunogenetic predisposition or environmental modifiers warrants further investigation. The chronological sequence of disease onset also appears to influence disease phenotype and severity. This observation parallels the psoriasis-to-psoriatic arthritis progression model, which includes an immune activation phase followed by a preclinical phase of joint inflammation, before clinical disease onset [22]. In the context of IBD, subclinical sacroiliac inflammation may be present for months or years before overt SpA develops. Indeed, sacroiliitis has been detected in 20% to 60% of patients with IBD in the absence of a formal SpA diagnosis, suggesting a potential preclinical phase of joint involvement [23].

Our findings highlight the therapeutic complexity that persists in this dual-diagnosis population. In our cohort, patients received an average of 2.8 lines of biologic therapies, and nearly 10% required combination bDMARDs, a rate significantly higher than in patients with SpA alone. This underlines both the refractoriness of disease in this population and the limited overlap in treatment efficacy between gut and joint inflammation. Recent data suggest that TNF inhibitors, although effective for IBD, may be insufficiently effective for concurrent axSpA, and vice versa. In a retrospective cohort study, approximately 50% of patients with IBD with newly developed SpA symptoms continued to report axial symptoms 1 year after initiating anti-TNF therapy [24]. A recent systematic review confirmed these findings, reporting frequent partial or nonresponse to anti-TNF agents in dual-diagnosis patients [25]. Regarding combination bDMARD therapy, the COMBIO French multicentre registry recently provided valuable insights. Among 143 patients receiving dual biologics, most had either CD (63.6%) or axSpA (37.7%), and about half were treated for a single disease, predominantly IBD [26]. Notably, the most common combination involved vedolizumab (anti-integrin, gut-specific) with an anti-TNF agent, underscoring a strategy wherein nonoverlapping mechanisms of action are leveraged to control disease in both organs.

The ‘difficult-to-treat’ (D2T) concept, well-established in rheumatoid arthritis [27], has recently been adapted for SpA [14]. In our study, nearly 32% of patients fulfilled D2T criteria—defined by the failure of ≥2 b/tsDMARDs with different mechanisms of action, in the context of persistent disease activity. This prevalence is substantially higher than the ∼9% reported in large national databases for patients with SpA alone [14,28,29], further supporting the notion that codiagnosis is associated with increased therapeutic resistance, at least on the rheumatological front. From a gastroenterological perspective, our dual-diagnosis cohort also exhibited higher clinical activity scores and lower remission rates, despite treatment [30]. These findings suggest that IBD activity may also be more difficult to control when SpA is present, potentially due to shared inflammatory pathways, higher systemic inflammation, or limitations in therapeutic choices imposed by the need to address both diseases concurrently.

In our cohort, the meantime interval between the 2 diagnoses was relatively short—2.1 years when SpA occurred first and 3 years when IBD occurred first. These findings suggest a close temporal proximity between the 2 disease onsets, supporting the hypothesis of a shared pathogenic continuum rather than 2 independent entities. Importantly, our data challenge the classical assumption that gut inflammation invariably precedes joint disease. In a substantial proportion of cases, joint damage preceded digestive symptoms, highlighting the need to systematically evaluate subclinical gut inflammation in patients with SpA, and vice versa.

Several limitations of this study must be acknowledged. First, the retrospective and monocentric design introduces potential biases in data collection and patient selection. The relatively small sample size limits the statistical power for subgroup analyses, although, to our knowledge, this represents one of the largest cohorts to date of patients with a confirmed dual diagnosis of SpA and IBD. Second, the chronology of diagnoses may not fully reflect the chronology of symptom onset, particularly in cases where subclinical or misattributed symptoms preceded the formal diagnosis. For instance, musculoskeletal complaints may have been under-recognised in patients initially managed for IBD, while subtle digestive symptoms may have been overlooked in patients with early SpA. Nevertheless, all patients in this study were recruited from a tertiary centre, where comprehensive and systematic screening for both extramusculoskeletal and extradigestive manifestations is routinely performed, thereby minimising this limitation. Third, the high proportion of patients requiring combination bDMARDs and fulfilling D2T criteria may reflect a referral bias linked to our setting as a tertiary care centre, with likely overrepresentation of severe or refractory cases.

## Conclusion

Patients with a dual diagnosis of SpA and IBD represent a distinct and challenging subgroup, with more severe disease, higher therapeutic burden, and frequent need for combined biologics. The sequence of onset influences phenotype, suggesting different disease trajectories. Our findings highlight the need for tailored strategies and further prospective studies to better understand and manage this complex population.

## Funding

None.

## Competing interests

None.

## Patient consent for publication

Not applicable.

## Ethics approval

Patients received information about the use of their medical data and did not object to the study before its start in accordance to the local Good Clinical Practice guidelines and with the Declaration of Helsinki. All participants were provided informed consent during the study. Clinical Research and Innovation Department of the Besançon University Hospital approved the experimental protocol. As a retrospective study, ethical review and approval were not required for the study in accordance with the local legislation and institutional requirements.

## Provenance and peer review

Not commissioned; externally peer reviewed.

## Data availability statement

The datasets used and/or analysed during the current study are available from the corresponding author on reasonable request.

## CRediT authorship contribution statement

**Juliane Michel:** Writing – review & editing, Writing – original draft, Visualization, Validation, Methodology, Investigation, Formal analysis, Data curation, Conceptualization. **Clément Prati:** Writing – review & editing, Writing – original draft, Visualization, Validation, Supervision, Methodology, Investigation, Funding acquisition, Formal analysis, Conceptualization. **Lucine Vuitton:** Writing – review & editing, Visualization, Validation, Methodology, Funding acquisition, Conceptualization. **Charline Vauchy:** Writing – review & editing, Visualization, Validation, Project administration, Methodology, Investigation. **Eric Toussirot:** Writing – review & editing, Visualization, Validation, Methodology. **Frederic Mauny:** Writing – review & editing, Visualization, Validation, Methodology, Conceptualization. **Marc Puyraveau:** Writing – review & editing, Visualization, Validation, Software, Formal analysis. **Mickaël Chouk:** Writing – original draft, Visualization, Validation, Investigation, Data curation. **Olivier Fakih:** Writing – review & editing, Writing – original draft, Visualization, Validation, Methodology, Investigation, Data curation, Conceptualization. **Daniel Wendling:** Writing – review & editing, Visualization, Validation, Methodology, Data curation, Conceptualization. **Frank Verhoeven:** Writing – review & editing, Writing – original draft, Visualization, Validation, Supervision, Project administration, Methodology, Investigation, Formal analysis, Data curation, Conceptualization.
